# Recombinant Tula hantavirus shows reduced fitness but is able to survive in the presence of a parental virus: analysis of consecutive passages in a cell culture

**DOI:** 10.1186/1743-422X-2-12

**Published:** 2005-02-22

**Authors:** Angelina Plyusnina, Alexander Plyusnin

**Affiliations:** 1 Haartman Institute, Department of Virology, University of Helsinki POB 21, FIN-00014, Helsinki, Finland

## Abstract

Tula hantavirus carrying recombinant S RNA segment (recTULV) grew in a cell culture to the same titers as the original cell adapted variant but presented no real match to the parental virus. Our data showed that the lower competitiveness of recTULV could not be increased by pre-passaging in the cell culture. Nevertheless, the recombinant virus was able to survive in the presence of the parental virus during five consecutive passages. The observed survival time seems to be sufficient for transmission of newly formed recombinant hantaviruses in nature.

## Background

Recombination in RNA viruses serves two main purposes: (i) it generates and spreads advantageous genetic combinations; and (ii) it counters the deleterious effect of mutations that, due to the low fidelity of viral RNA polymerases and lack of proofreading, occur with high frequency [1]. The purging function is, naturally, attributed to the homologous recombination (HRec), i.e. recombination between homologous parental molecules through crossover at homologous sites. HRec was first described for the positive-sense RNA viruses [2,3] and subsequent studies lead to the widely accepted copy-choice model [4]. HRec was later shown to occur in rotaviruses thus adding double-stranded RNA viruses to the list of viruses capable of recombination [5]. Negative-sense RNA viruses that occupy the largest domain in the virus kingdom until recently were known to undergo non-homologous recombination only, forming either defective genomes, like polymerase "mosaics" of influenza A virus DI-particles [6] and "copy-backs" of parainfluenza virus [7] or hybrids between viral and cellular genes [8] or between different viral genes [9]. The first evidence for HRec in a negative-sense RNA virus has been obtained on hantaviruses [10,11].

Hantaviruses (genus *Hantavirus*, family *Bunyaviridae*) have a tripartite genome comprising the L segment encoding the RNA-polymerase, the M segment encoding two external glycoproteins, and the S segment encoding the nucleocapsid (N) protein [12]. Hantaviruses are maintained in nature in persistently infected rodents, each hantavirus type being predominantly associated with a distinct rodent host species [13]. When transmitted to humans, some hantaviruses cause hemorrhagic fever with renal syndrome or hantavirus pulmonary syndrome, whereas other hantaviruses are apathogenic [14,15]. Persistent infection in natural hosts allows for the simultaneous presence of more than one genetically distinct hantavirus variant in the same rodent. This may result in hantavirus genome reassortment [16,17] or recombination, as proposed in the above-mentioned study of Sibold *et al *[10] who showed a mosaic-like structure of the S RNA segment and the N protein of Tula hantavirus (TULV). Most recently, we have shown transfection-mediated rescue of TULV with recombinant S segment, in which nt 1–332 originate from the cell culture isolate Moravia/Ma5302V/94 (or TULV02, for short) [18], nt 369–1853 originate from the strain Tula/Ma23/87 [19], and nt 333–368, that are identical in both variants, can be of either origin. Both M and L segments of the recombinant virus (recTULV) originate from TULV02 [11]. RecTULV was functionally competent but less competitive than TULV02. One reason for the observed lower fitness of the recTULV might be that it was generated in the presence of the wt variant, with which it has to compete, and thus not given enough time to to establish a well balanced, mature quasi-species population. We, therefore, decided to compare fitness of TULV02 with that of recTULV that underwent several passages in cell culture.

## Results and discussion

**Figure 1:**
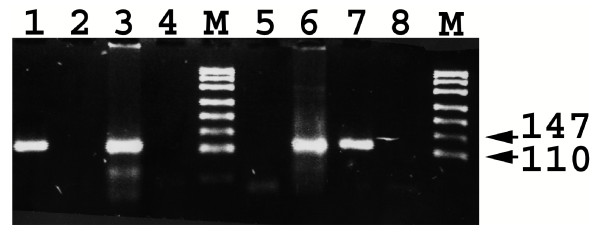
**Checking of specificity of RT-PCRs for the wt and the recombinant S RNA segments. **Lines 1–3: products of RT-PCR with primers VF738 and VR855 on RNA from cells infected with TULV02 (line 1), on RNA from cells infected with the recTULV (line 2) and on the mechanical mixture of both RNA preparations (line 3). Lines 5–7: the corresponding products of RT-PCR with primers RECF738 and RECR855. Lines 4 and 8 show negative controls. M, molecular weight marker; bands of 147 and 110 bp are indicated by arrows.

**Figure 2:**
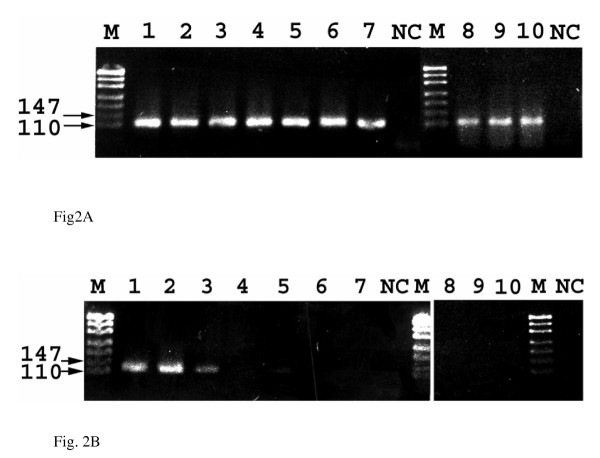
**Monitoring of wt and recS-RNA during sequential passages of the mixture of TUL02 and recTULV. A. **PCR-amplicons (118 bp), obtained in RT- PCR with the primers VF738 and VR855 (specific for the wt virus) on RNA from infected cells collected on passages 1 to 10. **B. **PCR-amplicons (118 bp), obtained in RT- PCR with the primers RECF738 and RECR855 (specific for the recombinant virus) on RNA from infected cells collected on passages 1 to 10. NC, negative controls. M, molecular weight markers; bands of 147 and 110 bp are indicated by arrows.

First, we designed RT-PCR primers able to discriminate between non-recombinant (V-type) and recombinant (REC-type) types of TULV S RNA. The resullts presented in Fig. 1 show that the primer pairs designed to generate the 118 bp- long products from either V-type or REC-type S RNA amplified, indeed, homologous sequences only, whether these were taken along (lines 1 and 6) or mixed with the heterologous sequences (lines 3 and 7). Using the two specific RT-PCR conditions, the presence of V-type and REC-type S RNA was monitored on ten sequential passages of the mixture of TULV02 and RecTULV5 variants (Fig. 2). S RNA of V-type was seen on all passages (Fig. 2A, lines 1–10). In contrast, S RNA of REC-type, was detected up to the fifth passage (Fig. 2B, lines 1–5), and then disappeared (Fig. 2B, lines 6–10). An alternative approach to check the presence of the two different types of S RNA using specific primer pairs at the stage of nested PCR gave exactly the same result. The V-type S RNA was detected during all ten passages while the REC-type totally disappeared after the 5^th ^passage (data not shown). These data confirmed our earlier observation [11] that the transfection-mediated HRec yields functionally competent and stable virus, recTULV. The purified and pre-passaged recombinant virus, however, presented no real match to the original cell adapted variant, TUL02, it terms of fitness. Taking into account that the *in situ *formed recombinant S RNA disappeared from the mixture after four passages [11], one should conclude that the lower competitiveness of the recombinant virus seen earlier did not result from its "immature" status. When, under similar experimental settings, TUL02 has been passaging in the presence of another isolate, TULV/Lodz, none of the two viruses was able to establish a dominance during ten consecutive passages (Plyusnin et al., unpublished data).

Although relatively short, the observed survival time of the recTULV in the presence of the original variant TUL02 seems to be sufficient for transmission of a recombinant virus, in a hypothetical *in vivo *situation, from one rodent to another. If transmission is performed in a sampling-like fashion – and this seems to be the case for hantaviruses [13] – the recombinant would have fair chances to survive. The existence of wt recombinant strains of TULV [10] supports this way of reasoning. Evidence for the recombination in the hantavirus evolution continues to accumulate [20,21].

**Figure 3:**
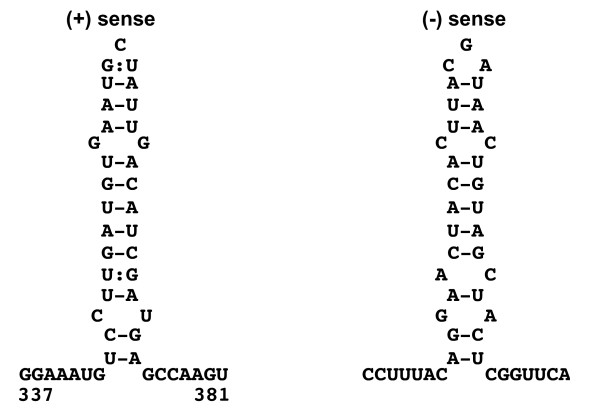
Hairpin-like structures predicted for the recombination "hot-spot" in the plus- and minus- sense S RNA of TULV.

The genetic swarm of S RNA molecules from the recTULV is represented almost exclusively by the variant with a single break point located between nt332 and nt368. The proportion of the dominant variant is larger in the passaged recTULV (13 of 14 cDNA clones analyzed, or 93%) than in the freshly formed mixture of recS RNAs (12 of 20 cDNA clones, or 60%) [11]. Thus, recTULV already represents a product of a micro-evolutionary play, in which the best-fit variant has been selected from the initial mixture of recS RNA. Whether this resulted from higher frequency of recombination through the "hot-spot" located between nt332 and nt368 or from the swift elimination of all other products of random recombination due to their lower fitness (the situation reported for polio- and coronaviruses [22,23]), or both, remains unclear. We favor the first explanation as the modeling of the S RNA folding suggests formation of a relatively long hairpin-like structure within the recombination "hot-spot" (Fig. 3). Secondary structure elements of this kind, which might present obstacles for sliding of the viral RNA polymerase along the template, were suggested as promoters for the template-switching in the early studies on polioviruses [22] and considered a crucial prerequisite for recombination [25,24]. The hairpin in TULV plus-sense S RNA (Fig. 3) is formed by the almost perfect inverted repeat that includes nt 344 to 374. In the minus-sense RNA, the structure is slightly weaker due to the fact that two non-canonical G:U base pairs presented in the plus-sense RNA occur as non-pairing C/A bases in the minus-sense RNA. Interestingly, in Puumala hantavirus, a hairpin-like structure formed by a highly conserved inverted repeat in the 3'-noncoding region of the S segment seems to be involved in recombination events, leading, however, to the deletion of the hairpin-forming sequences (A. Plyusnin, unpublished observations). The role of RNA folding in hantavirus recombination awaits further investigation.

## Conclusion

The data presented in this paper show that the recTULV presents no real match to the original cell adapted variant and that the lower fitness of the recombinant virus can not be increased by pre-passaging in cell culture. The observed survival time of the recTULV in the presence of the parental virus seems to be sufficient for transmission of newly formed recombinant hantaviruses in nature.

## Methods

### Recombinant TULV

RecTULV (clone 5) was purified from the mixture it formed with the original variant, TULV02, using two consequent passages under terminal dilutions [11]. After the purification, recTULV underwent three more passages, performed under standard conditions, i.e. without dilution. The presence of recS-RNA on the passages was monitored by RT-PCR and the isolate appeared to have a stable genotype (data not shown). RecTULV formed foci similar in size to those of the original variant and grew to the titers 5 × 10^3 ^– 10^4 ^FFU/ml.

### Competition experiments

Vero E6 cells (5 × 10^6 ^cells) were infected with the 1:1 mixture of recTULV and TULV02, approximately 10^4 ^FFU altogether. After 7–12 days the supernatant (~20 ml) was collected and RNA was extracted from the cells with TriPure™ isolation reagent, Boehringer Mannheim. Aliquots (2 ml) of the supernatant were used to infect fresh cells; the rest was kept at -70°C. The following nine passages were performed in the same way.

### Reverse transcription (RT), polymerase chain reaction (PCR) and sequencing

RT was performed with MuLV reverse transcriptase (New England Biolabs); for PCR, AmpliTaq DNA polymerase (Perkin Elmer, Roche Molecular Systems) was used. To monitor the presence of TULV S RNA on passages, RT-PCR was performed with primers VF738 (5'GCCTGAAAAGATTGAGGAGTTCC3'; nt 738–760) and VR855 (5'TTCACGTCCTAAAAGGTAAGCATCA3'; nt 831–855). To monitor the presence of recTULV S RNA, RT-PCR was performed with primers RECF738 (5'GCCAGAGAAGATTGAGGCATTTC3'; nt 738–760) and RECR855 (5'TTCTCTCCCAATTAGGTAAGCATCA3'; nt 831–855). All four primers were perfect matches to the homologous sequences; to the heterologous sequences, the forward primers have five mismatches while the reverse primers have six. Alternatively, complete S segment sequences of both variants of TULV were amplified using a single universal primer [19] and then either of the two pairs of primers was used in nested PCR. Authenticity of the PCR amplicons was confirmed by direct sequencing using the ABI PRISM Dye Terminator Sequencing kit (Perkin Elmer Applied Biosystems Division).

## Competing interests

The author(s) declare that they have no competing interests.

## Authors' contributions

AngP participated in the design of the study, carried out the experiments and helped to draft the manuscript. AlexP participated in the design of the study and drafted the manuscript. Both authors read and approved the final manuscript.
